# Thrills, chills, frissons, and skin orgasms: toward an integrative model of transcendent psychophysiological experiences in music

**DOI:** 10.3389/fpsyg.2014.00790

**Published:** 2014-07-23

**Authors:** Luke Harrison, Psyche Loui

**Affiliations:** Department of Psychology, Wesleyan UniversityMiddletown, CT, USA

**Keywords:** chills, frissons, emotion, music, review

## Abstract

Music has a unique power to elicit moments of intense emotional and psychophysiological response. These moments – termed “chills,” “thrills”, “frissons,” etc. – are subjects of introspection and philosophical debate, as well as scientific study in music perception and cognition. The present article integrates the existing multidisciplinary literature in an attempt to define a comprehensive, testable, and ecologically valid model of transcendent psychophysiological moments in music.

## DEFINITIONS AND SCOPE

The present article is about *that* moment when music resonates so deeply and viscerally as to elicit a physical, bodily response. In trying to describe and test this sensation, we will attempt to clarify the terminology and elaborate on some major pieces of evidence regarding the types of musical movements that elicit transcendent physical experiences. The relevant literature reviewed here is particularly interesting for its necessarily multidisciplinary nature (with inroads into neuroscience, psychology, ethnomusicology, and music analysis) as well as its unavoidable subjectivity in defining these intensely personal experiences.

We begin by examining the murky, but understatedly consequential issue of nomenclature: what is a transcendent, psychophysiological moment of musical experience, and how does its lexical treatment fit into popular and academic discourse? How have researchers described this sensation thus far? Which terms work and which fall short? To answer these questions, we draw from the fields of cognitive neuroscience, phenomenology, psychology, and ethnomusicology, each of which comprises a corollary component to the study of music and emotions.

Having arrived at a satisfactory operational definition of musical frissons, we will transition into a less abstract discussion of the sensation, roughly dividing its manifestations into the physical and the socio-cultural, interspersed with their respective relations to the emotional. With regard to physical responses, we will attempt to provide a taxonomy of highly prevalent psychophysiological responses to music (Craig, 2005; Guhn et al., 2007; Hodges, 2011). In doing so, we will present literature linking the intensity of psychophysiological responses to that of emotional responses (Sloboda, 1991; Panksepp, 1995; Huron, 2006; Koelsch, 2010; Gabrielsson, 2011), but will ultimately attempt to unpack the ontological root of musical emotion and problematize monodirectional routes of causation (Panksepp, 1995; Levinson, 2000) between the physical and the (perceived) emotional. We will also briefly discuss the neural substrates of transcendent moments of musical experience, with a focus on their interactions with motivation and reward systems. We will conclude by suggesting future approaches in selection of musical stimuli used to elicit human bodily responses to music.

## CHILLS, THRILLS, AND FRISSON

So what is a transcendent, psychophysiological moment of musical experience? In examining this question, one might begin by considering a broad, quasi-phenomenological framework such as that proposed by Gabrielsson (2011). He terms these moments “Strong Experiences with Music (SEM),” based loosely on Maslow’s “Peak Experience” (Maslow, 1962). The criteria for these SEMs include distinctiveness, ineffability, existential, or transcendental feelings, and, poignantly, physical or quasi-physical sensations and powerful emotions. The psychophysiological experiences most reported in Gabrielsson’s (2011) study were tears (24% of participants), chills/shivers (10%), and piloerection, or gooseflesh (5%). While the use of strong experiences with music provides a verbal framework that succeeds in its resistance to oversimplifying ecologically valid experiences, it is resistant to generalization and therefore untestable other than through the paradigm of self-report.

The prevailing terminology in mainstream musical and psychophysiological discourse has tended toward hyperspecificity, to the extent that it is often reductive. The most popular terms in both academic and popular discourse are “chills” and “thrills” (Huron and Margulis, 2011), often used interchangeably (Grewe et al., 2007; Huron and Margulis, 2011). Both aim at identifying significant and easily testable parts of the transcendent moments at hand, but both suffer from a lack of operative, institutional consensus.

“Chills,” the most popular term (Huron and Margulis, 2011), enjoys a ubiquity in popular culture that has left it particularly open to a variety of definitions. There is some consensus that chills entail a rapidly spreading, tingling feeling, but additional traits remain in dispute. Some scholars concretely include gooseflesh in their concept of chills (Panksepp, 1995; Guhn et al., 2007), while some state that gooseflesh is merely a common companion of chills (Grewe et al., 2007; Hodges, 2009), and still others claim that gooseflesh is only induced in approximately 50% of all chill responses (Craig, 2005). Even if we reduce chills to the tingling sensation alone, there remains a lack of consensus regarding its location in the body. Grewe et al. (2007, p. 300) included a participant’s chills in their analysis only if the she/he reported gooseflesh and/or ‘shivers down the spine,’ but 2 years earlier, Craig (2005, p. 278) found that his participants were most likely to experience chills in their arms, while less then half felt anything in their spine. A similar dispute can be found regarding the inclusion of certain psychophysiological measures, most notably skin conductance responses (SCR). Grewe et al. (2007), again, cite SCR as a necessary criterion for inclusion in their analysis of chills, while many other scholars merely consider them a correlate of chills (Craig, 2005; Guhn et al., 2007).

Another popular descriptor of this sensation, “thrills,” may provide additional clarity to “chills” in a few crucial ways. Unlike “chills,” “thrills” is defined, not only as a “shudder or tingling throughout the body,” but as one that also includes emotional intensity (Oxford English Dictionary, Frisson, n.d.). It sidesteps some of the conflicts surrounding “chills,” perhaps for no other reason than that it is less often used. However, the cultural associations conjured by “thrills” are complex and render the term problematic. To a cognitive scientist, a “thrill” may be a tingling sensation, but to the lay participant of a study, the word “thrill” retains a non-physiological meaning that may prove impossible to entirely subvert. This issue already seems to have manifested itself experimentally, as Goldstein (1980) studied this sensation as a “thrill” and found it to be more often elicited by happy music than by sad music, while Panksepp (1995) studied the same phenomenon as a “chill” and found it more often elicited by sad music than by happy music (Panksepp, 1995, p. 194).

It is this issue of cultural association that has disqualified the oft-referenced, but rarely used term, “skin orgasm,” despite its uniquely accurate description of the spectrum of musically induced emotional phenomena (Panksepp, 1995). The term implies a pleasurable sensation that is paradoxically both universal and variable. It affects different parts of the body depending on the person and circumstances of induction, and retains similar sensory, evaluative, and affective biological and psychological components to sexual orgasm (Mah and Binik, 2001). Furthermore, transcendent, psychophysiological moments of musical experience have been shown to incorporate the same neural reward pathways as such visceral pleasures as food and sex (Blood and Zatorre, 2001). However, the term has not gained scholarly traction, presumably because of its complicated associations with sexual conventions, which differ drastically between cultures, regions, and people. As theoretically accurate as “skin orgasm” may be, it seems unlikely that most potential participants (primarily college students) in studies on the phenomenon would be able to disassociate themselves sufficiently from their individual relationships with sexual orgasm to subvert their own biases.

This leaves us with one highly prominent term left to cover: frisson, described by Huron and Margulis (2011, p. 591) as “a musically induced affect that shows close links to musical surprise” and is associated with a “pleasant tingling feeling,” raised body hairs, and gooseflesh. One might supplement this domain-specific definition with one from the Oxford English Dictionary (Thrill, n.d.), simply, “an emotional thrill.” “Frisson” may be the most accurate and usable term because it integrates emotional intensity with verifiable tactile sensations not localized to any one region of the body. Its relative specificity and obscurity in popular culture allow it to avoid loaded cultural association; furthermore, it does not have the thermal priming potential of the cold-inducing, “chills.”

In adopting the term “frisson” we would, however, recommend that the term be expanded to include other perceptible but non-dermal reactions such as tears, lump-in-the-throat sensations, and muscle tension/relaxation (Sloboda, 1991; Hodges, 2011) to form a more integrative, generalizable frisson concept^1^.

## THE IMPORTANCE OF CONTEXT

In trying to explain musical frisson, the philosophical literature has addressed varieties of musical qualia, or musical consciousness. Goguen (2004) argues that key aspects of the musical consciousness involve enactment and social context (Goguen, 2004). Drawing on cultural anthropological work on the musical induction of altered states of consciousness, Bicknell (2009) also notes that the supposition of a simple causal relationship between a musical feature (e.g., rhythm) and trance (e.g., due to the effects of rhythm on the central nervous system) is problematic, as musical features can have different effects in different cultures and within varying social contexts. This critical role of social and cultural context places the act of music listening in an intrinsically social setting. In some contexts, the need for social bonding may give rise to strong emotional responses to music (Bicknell, 2007).

The social nature of music making fits with evolutionary theories of music as a transformative technology of the mind (Patel, 2008; Altenmüller et al., 2013). According to these theories, human-made sounds that originated as an affective communication system may have gradually honed the human mind into an entity that treats music an esthetic experience, including peak experiences. This ability confers a common intuitive grasp of the sublime (Konečni, 2010), resulting in an esthetic communicative power that may have shaped the evolution of music in its myriad contexts, and may also have provided the added advantage for music as a safe playground for new auditory experiences (Altenmüller et al., 2013).

Studies from ethnomusicology are also relevant here, as they provide contexts in which one may place the discussion of musical frisson. In her study of music and trance, Becker (2004) emphasizes that many cultures conceive of music as an integrative, full-body phenomenon. Some Gospel devotees report being so overcome by musically induced spiritual ecstasy that they have entered a quasi-comatose physical state (Jungr, 2002, p. 111), while North Indian and Pakistani Sufis have long considered there to be an erotic experiential dimension to deep music listening (Becker, 2004, pp. 61–62). In fact, many regional West African languages^2^ do not have a word for music as a solely auditory phenomenon. Rather, any proper translation of “music” necessarily includes a strong choreographic element and active communal participation, whose musical synchrony depends on oral transmission or collective feel (Nketia, 1974; Chernoff, 1981; Agawu, 1995; Agawu, 2003). Given this cross-cultural perspective, it becomes clear that music involves, as Levinson (2000, p. 73) describes, the “‘whole person’… cognitive, emotional, sensational, and behavioral at once.” In contrast, the more traditional view of music as a mental phenomenon with some localized psychophysiological correlates may be overly reductive.

If one goal of studying musical frisson is to examine the body’s reactions to transcendent auditory stimuli that are idiomatically embedded in sound art (music), then it is important to avoid erroneous universalism, especially when this universalism is implicitly derived from a dualist view (Becker, 2004, p. 6) wherein the body is subordinate to the mind, rather than its simultaneous and interactive embodiment.

This implicit mind–body hierarchy is pervasive in studies of music emotion, which assume that frissons are the effect of emotions, rather than part of their cause or an unrelated but simultaneous phenomenon. For instance, Panksepp (1995) observed that listeners reported higher instances of frisson during sad music than during happy music. From this it was concluded that frissons are more readily elicited by sadness than by happiness. While this conclusion may be justified, the results do not rule out the alternative hypotheses that the musical attributes of sad music (slow tempo, descending melisma, etc.) might be more likely to elicit both frissons and sadness concurrently. Another alternative hypothesis is that participants’ psychophysiological construction of “chills” might include phenomena such as tears and cold, also associated with sadness.

## COMPONENTS OF EMOTIONAL RESPONSES TO MUSIC

Although a very strong relationship exists between musical frisson and perceived emotion (Panksepp, 1995; Blood and Zatorre, 2001; Huron, 2006; Juslin and Västfjäll, 2008; Lundqvist et al., 2009; Salimpoor et al., 2009; Juslin, 2013) the interplay between these emotions and frisson is complex. Juslin (2013, p. 240) proposes a revised eight-pronged model of emotions elicited by music, which incorporates a variety of social, autobiographical, psychophysiological, and psychological factors. These eight “mechanisms” are (1) brainstem reflexes, (2) rhythmic entrainment, (3) evaluative conditioning, (4) contagion, (5) visual imagery, (6) episodic memory, (7) musical expectancy, and (8) esthetic judgment. Although all of these mechanisms are interrelated, the present article will focus on mechanisms 1, 3, 4, and 7, as they are most relevant to frisson.

The first mechanism, brainstem reflexes, primarily concerns arousal of the autonomic nervous system (ANS). Activation in the ANS has been shown to spike at the onset of loud, very high or low frequency, or rapidly changing sounds. Notably, these properties, as well increased heart rate, SCR, and respiratory depth, three pillars of ANS arousal, have all consistently been shown to correspond with the onset of frisson (Blood and Zatorre, 2001; Craig, 2005; Guhn et al., 2007). The connection between frisson and the ANS is further bolstered by Goldstein’s (1980) study, which effectively blocked the musical induction of frisson using an opioid antagonist.

The third mechanism, evaluative conditioning, involves the learning of paired associations between music (conditioned stimulus), and physical sensations of frisson (unconditioned stimulus) to produce general frisson (unconditioned response) followed by musical frisson (conditioned stimulus). Esthetic appraisal follows this conditioning process. While musical frisson may be learnable from such a process, it remains to be determined to what extent this evaluative response reflects moments within the musical structure *per se*, or whether the emotional component is more proximally garnered from autobiographical associations with contextual musical stimuli.

Juslin’s (2013) fourth mechanism, emotional contagion, concerns one’s ability to determine an expressed emotion from a stimulus (in this case auditory) and then mirror that emotion empathically. For instance, if we hear sad music, we are able to recognize that sadness and allow ourselves to feel sad, despite the absence of a human, verbal expression of sadness. This mechanism relates to frisson if we conceive of frisson as a contributor to perceived emotional intensity (Blood and Zatorre, 2001; Huron, 2006; Juslin and Västfjäll, 2008; Juslin, 2013). Emotional contagion may determine the emotional content of music, while the perceived intensity of that emotion is moderated by frisson.

The seventh mechanism in Juslin’s (2013) model, musical expectancy, refers to emotions elicited when one’s explicit or implicit expectations are violated. The idea that musical emotions depend on expectations is likely the most extensively theorized and researched of the eight mechanisms (see Meyer, 1956). Expectancy violations (e.g., harmonic, rhythmic, and/or melodic violations) are strongly correlated to the onset of musical frisson, such that some level of violated expectation may be a prerequisite (Sloboda, 1991; Huron, 2006; Steinbeis et al., 2006). The use of musical expectancy as a reliable frisson-inducer has provided researchers with a viable, if reductionist, scientific approach in which peak emotional experiences may be identifiable and even inducible, via the systematic manipulation of expectancy in music.

## NEUROBIOLOGICAL MECHANISMS

The philosophical problem of frisson as musical qualia can also be approached from the perspectives of psychology and neuroscience (Cochrane, 2010). Peak musical emotional experiences, including those which elicit musical frisson, take place in two anatomically distinct areas of the dopaminergic reward system: the caudate, which activates in the anticipatory moments preceding one’s emotional peak, and the nucleus accumbens, which activates during the release immediately after this peak (Salimpoor et al., 2011). In addition, the functional and structural connectivity between auditory areas and emotional and reward processing systems is a successful predictor of frisson (Salimpoor et al., 2013; Sachs et al., under review), which suggests that frisson involves not only single, modular reward-processing regions, but rather a network of both reward and emotional processing regions functioning in concert with auditory-motor activity.

Blood and Zatorre (2001) showed a similar pattern of results with their focus on neural reward systems in their landmark 2001 PET study on musical frisson. They found that listening to frisson-inducing music (relative to a control piece) corresponded with cerebral blood flow (CBF) changes to the midbrain, left ventral striatum, bilateral amygdala, left hippocampus, and ventromedial prefrontal cortex. These patterns may reflect a “craving” reflex similar to that surrounding responses to food, sex, and drugs of abuse (p. 11823). It is possible, then, that the reason we develop such affinity for frisson-inducing music is that once we experience musical frisson, we develop a dopaminergic anticipation for its return, effectively becoming slightly addicted to the musical stimulus.

Blood and Zatorre (2001) also found positive correlations between the reported intensity of frisson responses and activity in distributed brain regions. These include paralimbic areas (bilateral insula, right orbitofrontal cortex), regions associated with ANS arousal (thalamus, anterior cingulate), and motor areas (cerebellum, supplementary motor area). Integrating the functions of these regions may explain listeners’ occasional muscular reactions (tension/relaxation) to music, as well as pleasurable responses to psychophysiological reactions such as SCR and heart rate fluctuation.

In addition to changes in activity in the brain regions above, recent research has suggested that highly pleasurable music may elicit greater connectivity between regions. Salimpoor et al. (2013) found positive correlations between valuations of unfamiliar musical stimuli and connectivity between auditory and reward-processing areas. Sachs et al. (under review) identified associations between heart rate during frisson and white matter volume connecting areas implicated in auditory and socio-emotional processing. These results allude to a possible role of musical frisson as a functional integrator of sensory processing with socio-emotional and psychophysiological control systems in the brain.

## MUSICAL FRISSON INDUCERS

Having established an integrative framework of frisson, we turn from its biological functions to ask what types of musical stimuli tend to induce frisson. In a classic study of music lovers’ most intense psychophysiological responses to music (Sloboda, 1991), Sloboda not only catalogd the individual musical stimuli recorded by his participants, but also their specific, corresponding psychophysiological reactions. Although these results may be affected by sampling and self-report biases, they do allude to a diversity of experience that may have been neglected by recent discourse in favor of more reductionist scientific measures.

Sloboda (1991, p. 114) found that the most common types of musical phrases to elicit frisson were chord progressions descending the circle of fifths to the tonic, melodic appogiaturas, the onset of unexpected harmonies, and melodic or harmonic sequences^3^. Other investigators subsequently pursued and expanded on these findings. Grewe et al. (2007) examined the effects of larger, musical structural elements on the induction of frisson. Their measure of frisson responses, although self-reported, took place in real time in a lab setting (participants were asked to press a button when they “got chills” while listening to music), and the experimenters chose the musical stimuli based on cultural prominence and genre representativeness (p. 299). They found that onsets of frisson were most likely to occur during peaks in loudness, moments of modulation, and works in which the melody occupied the human vocal register. The vast majority of studies on frisson that have incorporated music analysis, including retrospectives of Sloboda’s (1991) study (Huron, 2006, p. 282), have identified sudden dynamic leaps (mostly from soft to loud, though moves to extreme softness have occasionally been shown to elicit the same effect) as major catalysts for frisson (Panksepp, 1995; Guhn et al., 2007). These findings support brainstem reflexes and expectancy violation as two components of Juslin’s (2013) model reviewed above.

## CONCLUSIONS: THE NEED FOR BROADER CONTEXT

It is important to note that many scholars have tended to study frisson primarily through the lens of Western art music (classical music), as opposed to popular, folk, and/or “world music” genres (Sloboda, 1991; Levinson, 2000; Huron, 2006; Steinbeis et al., 2006; Gabrielsson, 2011). Although there is, of course, nothing wrong with the study of these undoubtedly reconstructive and ubiquitously influential classical genres, it is important to maintain an egalitarian perspective, as music that induces frisson can be found across most, if not all, cultures and genres. Therefore, to restrict our stimuli to Western classical music is to restrict the diverse contexts in which frisson may occur, thereby limiting the ecological validity of our claims. We understand, of course, that researchers are not entirely to blame for this institutional bias. These studies draw primarily from student populations, and music students tend to listen to more classical and less popular music than the general population. That being said, people are more likely to react physically to familiar music than to unfamiliar music (Panksepp, 1995; Pereira et al., 2011), so to favor Western classical music over other genres of stimuli, such as popular music or the music of one’s own culture, is to prioritize the opinions of Western classical music lovers over those of popular music lovers. Although the field has already begun to move in a broader musical direction (Craig, 2005; Grewe et al., 2007), a concerted effort should be made to test the potential for frisson induction across as many different genres as possible. Only then will we effectively approach a more nuanced view of the timbral, rhythmic, and cultural contexts that may relate to musical frisson.

This encompassment of nuance, of course, is a difficult goal. In music cognition, where many professional scientists are also amateur artists and performers, it seems particularly likely that one would find a scholarly community hyper-aware of the burden of laboratory constraints and of the disparity between experimental and real-life artistic stimuli and environments. For better or worse, musicians and music lovers do not divide along disciplinary lines, so in order to advance the science of music, one must let it occasionally concede some of its authority to an experiential, and phenomenological truth that represents music more than it does cognition. Future studies that acknowledge and respect individual differences in subjective experience may yield fruitful knowledge about the shared and unique experiential dimensions of musical frisson. In doing so, we might achieve a fuller view of cognitive and social behavior to the substantial benefit of an ever-growing musical neuroscience.

## Conflict of Interest Statement

The authors declare that the research was conducted in the absence of any commercial or financial relationships that could be construed as a potential conflict of interest.

## Footnotes

1 We are aware that this revision would undercut the word’s etymological and practical history, but perhaps such a change could be accomplished by qualifying that the expanded definition could only apply to, in Levinson’s (2000) words, “Musical Frissons.”

2 The most studied of these languages in the Western academy is Ewe, belonging to the ethnic group of the same name who originate in and around Northern Ghana. For more on the interaction between the Ewe language and West African constructions of music, see *African Rhythm: A Northern Ewe Perspective* (Agawu, 1995).

3 Tears were most likely to be elicited by appoggiaturas; shivers or gooseflesh by the onset of new harmonies.

## References

[B1] Agawu K. (2003). *Representing African Music: Postcolonial Notes, Queries, Positions*. New York: Routledge.

[B2] Agawu V. K. (1995). *African Rhythm: A Northern Ewe Perspective*. Cambridge: Cambridge University Press

[B3] Altenmüller E. Kopiez R. Grewe O. (2013). “Strong emotions in music: are they an evolutionary adaptation?” in *Sound–Perception–Performance* ed.Bader R. (Dordrecht: Springer), 131–156

[B4] Becker J. (2004). *Deep Listeners: Music, Emotion, and Trancing*. Bloomington, IN: Indiana University Press

[B5] Bicknell J. (2007). Explaining strong emotional responses to music: sociality and intimacy. *J. Conscious. Stud.* 14 5–23

[B6] Bicknell J. (2009). *Why Music Moves Us*. New York: Palgrave MacMillan 10.1057/9780230233836

[B7] Blood A. J. Zatorre R. J. (2001). Intensely pleasurable responses to music correlate with activity in brain regions implicated in reward and emotion. *Proc. Natl. Acad. Sci. U.S.A.* 98 11818–11823 10.1073/pnas.191355898 11573015 PMC58814

[B8] Chernoff J. M. (1981). *African Rhythm and African Sensibility: Aesthetics and Social Action in African Musical Idioms*. Chicago: Chicago University Press

[B9] Cochrane T. (2010). Music, emotions and the influence of the cognitive sciences. *Philos. Compass* 5 978–988 10.1111/j.1747-9991.2010.00337.x

[B10] Craig D. G. (2005). An exploratory study of physiological changes during “chills” induced by music. *Music. Sci.* 9 273–287

[B11] Frisson.(n.d.). *Oxford English Dictionary Online*. Available at: http://www.oed.com/view/Entry/74785 [accessed May 6, 2014].

[B12] Gabrielsson A. (2011). “Strong experiences with music,” in *Handbook of Music and Emotion: Theory, Research, Applications,* eds Juslin P. N. Sloboda J. A. (New York: Oxford University Press), 547–574 10.1093/acprof:oso/9780199695225.001.0001

[B13] Goguen J. A. (2004). Musical qualia, context, time and emotion. *J. Conscious. Stud.* 11 117–147

[B14] Goldstein A. (1980). Thrills in response to music and other stimuli. *Physiol. Psychol.* 8 126–129 10.3758/BF03326460

[B15] Grewe O. Nagel F. Kopiez R. Altenmüller E. (2007). Listening to music as a re-creative process: physiological, psychological, and psychoacoustical correlates of chills and strong emotions. *Music Percept.* 24 297–314 10.1525/mp.2007.24.3.297

[B16] Guhn M. Hamm A. Zentner M. (2007). Psychological and musico-acoustic correlates of the chill response. *Music Percept.* 24 473–484 10.1525/mp.2007.24.5.473

[B17] Hodges D. A. (2009). “Bodily responses to music,” in *The Oxford Handbook of Music Psychology* eds Hallam S. Cross I. Thaut M. (Oxford: Oxford University Press), 121–130

[B18] Hodges D. A. (2011). “Psychophysiological measures,” in, *Handbook of Music and Emotion: Theory, Research, Applications*, eds Juslin P. N. Sloboda J. A. (New York: Oxford University Press), 279–311

[B19] Huron D. (2006). *Sweet Anticipation: Music and the Psychology of Expectation*. Cambridge: The MIT Press

[B20] Huron D. Margulis E. H. (2011). “Music expectancy and thrills,” in, *Handbook of Music and Emotion: Theory, Research, Applications*, eds Juslin P. N. Sloboda J. A. (New York: Oxford University Press), 575–604

[B21] Jungr B. (2002). “Vocal expression in the blues and gospel,” in *The Cambridge Companion to Blues and Gospel Music* ed. Moore A. (Cambridge: Cambridge University Press), 102–115

[B22] Juslin P. N. (2013). From everyday emotions to aesthetic emotions: towards a unified theory of musical emotions. *Phys. Life Rev.* 10 235–266 10.1016/j.plrev.2013.05.008 23769678

[B23] Juslin P. N. Västfjäll D. (2008). Emotional responses to music: the need to consider underlying mechanisms. *Behav. Brain Sci.* 31 559–575 10.1017/S0140525X08005293 18826699

[B24] Koelsch S. (2010). Towards a neural basis of music-evoked emotions. *Trends Cogn. Sci.* 14 131–137 10.1016/j.tics.2010.01.002 20153242

[B25] Konecni V. J. (2010). Aesthetic trinity theory and the sublime. *Proc. Eur. Soc. Aesthet.* 2 244–264

[B26] Levinson J. (2000). Musical frissons. *Rev. Fr. Etud. Am.* 86 64–76

[B27] Lundqvist L. O. Carlsson F. Hilmersson P. Juslin P. N. (2009). Emotional responses to music: experience, expression, and physiology. *Psychol. Music* 37 61–90 10.1177/0305735607086048

[B28] Mah K. Binik Y. M. (2001). The nature of human orgasm: a critical review of major trends. *Clin. Psychol. Rev.* 21 823–856 10.1016/S0272-7358(00)00069-6 11497209

[B29] Maslow A. H. (1962). *Toward A Psychology of Being*. Princeton: Van Nostrand 10.1037/10793-000

[B30] Meyer L. B. (1956). *Emotion and Meaning in Music*. Chicago, IL: University of Chicago Press

[B31] Nketia J. H. K. (1974). *The Music of Africa*. New York: W. W. Norton

[B32] Panksepp J. (1995). The emotional sources of “chills” induced by music. *Music Percept.* 13 171–207 10.2307/40285693

[B33] Patel A. D. (2008). “Music as a transformative technology of the mind,” in *Proceedings of the symposium at Music: Its Evolution, Cognitive Basis, and Spiritual Dimensions,* Cambridge

[B34] Pereira C. S. Teixiera J. Figueiredo P. Xavier J. Castro S. L. Brattico E. (2011). Music and emotions in the brain: familiarity matters. *PLoS ONE* 6:e27241 10.1371/journal.pone.0027241 PMC3217963 22110619

[B35] Salimpoor V. N. Benovoy M. Larcher K. Dagher A. Zatorre R. J. (2011). Anatomically distinct dopamine release during anticipation and experience of peak emotion to music. *Nat. Neurosci.* 14 257–262 10.1038/nn.2726 21217764

[B36] Salimpoor V. N. Benovoy M. Longo G. Cooperstock J. R. Zatorre R. J. (2009). The rewarding aspects of music listening are related to degree of emotional arousal. *PLoS ONE* 4:e7487 10.1371/journal.pone.0007487 PMC2759002 19834599

[B37] Salimpoor V. N. van den Bosch I. Kovacevic N. McIntosh A. R. Dagher A. Zatorre R. J. (2013). Interactions between the nucleus accumbens and auditory cortices predict music reward value. *Science* 340 216–219 10.1126/science.1231059 23580531

[B38] Sloboda J. A. (1991). Music structure and emotional response: some empirical findings. *Psychol. Music* 19 110–120 10.1177/0305735691192002

[B39] Steinbeis N. Koelsch S. Sloboda J. A. (2006). The role of harmonic expectancy violations in musical emotions: evidence from subjective, physiological, and neural responses. *J. Cogn. Neurosci.* 18 1380–1393 10.1162/jocn.2006.18.8.1380 16859422

[B40] Thrill. (n.d.). *Oxford English Dictionary Online*. Available at: http://www.oed.com/view/Entry/74785? [accessed May 6, 2014].

